# Solid-State Synthesis of Poly(3',4'-dimethoxy-2,2':5',2"- terthiophene): Comparison With Poly(terthiophene) and Poly(3',4'-ethylenedioxy-2,2':5',2"- terthiophene)

**DOI:** 10.3390/molecules17078647

**Published:** 2012-07-23

**Authors:** Tursun Abdiryim, Ruxangul Jamal, Aminam Ubul, Ismayil Nurulla

**Affiliations:** Key Laboratory of Petroleum and Gas Fine Chemicals, Educational Ministry of China, College of Chemistry and Chemical Engineering, Xinjiang University, Urumqi 830046, China; Email: tursunabdir@sina.com.cn (T.A.); jruxangul@xju.edu.cn (R.J.); aminam520@gmail.com (A.U.)

**Keywords:** poly (terthiophene), dimethoxy substituents, solid-state oxidative polymerization

## Abstract

A new terthiophene monomer: 3',4'-dimethoxy-2,2':5',2"-terthiophene (TMT) was synthesized and characterized by ^1^H-NMR, ^13^C-NMR and FTIR. The solid-state oxidative polymerizations of TMT were performed in various ratios of oxidant (FeCl_3_) to monomer (TMT). The resulting polymers were characterized by ^1^H-NMR, FTIR, UV-vis-NIR, GPC, X-ray diffraction, CV, as well as TGA and conductivity measurements. The structure and properties of poly (TMT) were compared with those of polyterthiophene [poly(TT)] and poly (3',4'-ethylenedioxy-2,2':5',2"-terthiophene) [poly(TET)] prepared under the same polymerization conditions. After comparative analysis with poly(TT) and poly(TET), the effects of the dimethoxy substituent and FeCl_3_ on the structural and physicochemical properties of the poly(TMT)s were discussed in depth. The comparison suggested that the dimethoxy-substituted polymer did not display higher crystallinity, thermal stability, conductivity and electrochemical activity than ethylenedioxy substituted one. The results also showed that the effect of FeCl_3_ on poly(TMT) was similar that seen with the poly(TT), in which the oxidation degree, electrochemical activity and conductivity increased steadily with increasing [FeCl_3_]/[TT] ratio. Furthermore, the poly(TMT) and poly(TT) are mostly made up of dimers with a small amount of higher molecular weight components.

## 1. Introduction

Among the various conjugated polymers, poly(thiophene) and its derivatives (PTs) have become the focus of considerable interest due to a unique combination of original electronic properties, environmental stability, and structural versatility [1,2], but PTs suffer from the occurrence of undesired α,β and β,β'-couplings during polymerization, which deteriorate their properties [3,4]. Incorporation of the substituents into the thiophene ring or cyclization between the 3 and 4 positions of the thiophene ring is a convenient way for preparing perfectly stereoregular, long conjugated polymers by elimination of α,β and β,β'-couplings [4]. The terthiophene type compounds are interesting monomers for designing new PTs, because of their preexisting α,α'-linkages and lower oxidation potential than that of thiophene monomer [5,6,7,8,9]. Furthermore, these precursors can be polymerized either chemically or electrochemically to form stable electroactive polymers [5,6,7,8,9,10,11]. Recently, we have demonstrated a novel room-temperature solid-state oxidative method for polymerization of ethylenedioxy-substituted terthiophene (TET), and we have found that the solid-state polymerization was an effective method for terthiophene type monomers [12].

In this paper, we synthesized a new terthiophene derivative: 3',4'-dimethoxy-2,2':5',2"-terthiophene (TMT), in which the 3 and 4 positions of the internal thiophene ring was substituted by free methoxy groups. The structure of TMT is similar to that of TET, in which dimethoxy groups are taken together to form the ethylenedioxy bridge. However, the dimethoxy units in terthiophene have different electron-donating effects and dihedral angles between the planes of adjacent aromatic units, compared with the ethylenedioxy unit in terthiophene. Thus, it can be deduced that the structure and properties of resulting poly(TMT) polymer should be different from those of poly(TET) and unsubstituted poly(TT). Therefore, it is reasonable to undertake a comparative study of the structure and properties of a series of polyterthiophenes obtained from TMT, TT and TET under the same polymerization conditions to further investigate the effect of free dimethoxy groups and FeCl_3_ on the structure and properties of resulting polymers. In the present work, with the above aim in mind, a room-temperature solid-state polymerization method was applied for oxidative polymerization of the new terthiophene monomer TMT by varying the molar ratio of FeCl_3_ to the TMT. The structure and properties of poly(TMT) were compared with those of poly(TT) and poly(TET) prepared under the same polymerization conditions. The effects of the dimethoxy substituents and FeCl_3_ on the structural and physicochemical properties of the resulting polymers were discussed in depth after comparison of the respective FTIR, UV-vis-NIR, X-ray diffraction, CV, as well as TGA and molecular weight and conductivity measurements.

## 2. Results and Discussion

*2.1. ^1^* *H-NMR, FT-IR Spectra* *and Molecular Weight*

**Figure 1:**
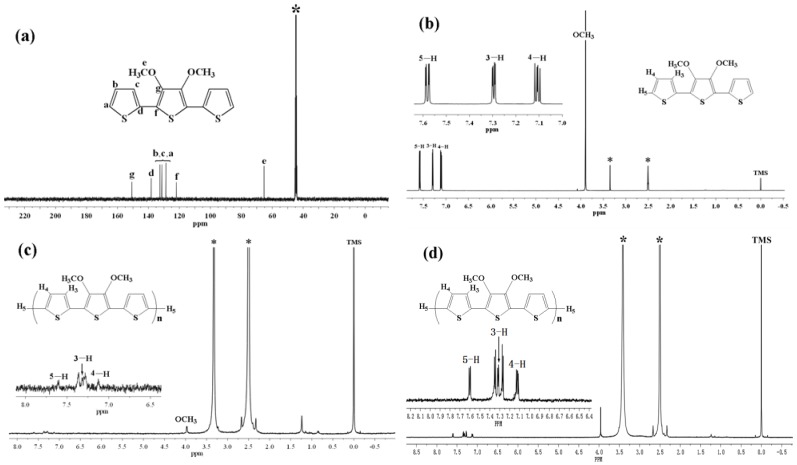
^13^C-NMR spectrum and ^1^H-NMR spectra of monomer and polymer: (**a**) ^13^C-NMR spectra of TMT in DMSO-d_6_; (**b**) ^1^H-NMR spectra of TMT in DMSO-d_6_; (**c**) poly (TMT)_3_ in DMSO-d_6_; (**d**) undoped poly (TMT)3 in DMSO-d_6_; Starred peaks comes from H_2_O and DMSO-d_6_.

Figure 1a represents the ^13^C-NMR spectrum of TMT in DMSO-d_6_, and Figure 1b–d represent the comparison of the ^1^H-NMR spectra of TMT and as-prepared and undoped poly(TMT)3 ([FeCl_3_]/[TMT] ratio of 8:1) in DMSO-d_6_. The ^13^C-NMR and ^1^H-NMR spectra of TMT show the typical resonance lines for this monomer, and their assignments are shown in Figure 1a,b. As depicted in Figure 1c,d, the ^1^H-NMR spectra of as-prepared and undoped poly (TMT)3 showed a peak in the aliphatic region at *δ* *~* 3.97 and five aromatic peaks in the *δ* *~* 7.13–7.60 range. Based on the assignment of ^1^H-NMR spectra of TMT, the *δ* *~* 3.97 peak is attributed to the O–CH_3_ protons [15]. The resonance peak at *δ* *~* 7.60 is attributed to the hydrogen atoms on the α-position of the terminal thiophene, and the resonance peaks at *δ* *~* 7.36, 7.13 are attributed to the hydrogen atoms on the β-position of the terminal thiophene, while the other two resonance peaks at *δ* *~* 7.36, 7.28 may arise from the inner units of the 2,5 disubstituted rings [12,16].

If one roughly estimates the integral intensity of five clear peaks at δ ~ 7.13–7.60 ppm of as-prepared and undoped poly (TMT)_3_ ([FeCl_3_]/[TMT] ratio of 8:1) in Figure 1c,d, it looks like the intensity of peaks, corresponding to each of H_5_, H_4_ and H_3_ protons at the polymer chain ends are more or less the same as the intensity of each peak attributed to the inner units of the 2,5-disubstituted rings. Furthermore, the ratio of surface integrals for the peaks at δ ~ 3.97 ppm and δ = 7.60 ppm is equal to 2, suggesting the poly(TMT) has a dimeric structure, containing in total six thiophene rings. This result is quite similar to the electrochemically synthesized poly(TET), which was found to be dimer of TET [12]. However, this was not confirmed by the GPC results shown in Table 1, which indicated a higher Mw ~ 3,400–3,600. This contradiction could result from overestimation of the Mw from GPC calibrated by PS standards, since the dimer has a rigid linear structure, while the standard is a flexible polymer-polystyrene. Therefore, we can conclude that the poly(TMT) is mostly a dimer of TMT with a small amount of higher molecular weight components (trimer or longer oligomers of TMT).

**Table 1:** The molecular weight, yield and conductivity of the polymers.

Polymers	[FeCl_3_]/[monomer]	Mn	Mw	PDI	Yield/%	Conductivity
poly(TMT)1	2:1	3,400	4,300	1.21	47%	1.2 × 10^−3^ S/cm
poly(TMT)2	4:1	3,600	4,400	1.22	73%	3.2 × 10^−3^ S/cm
poly(TMT)3	8:1	3,500	4,400	1.26	81%	4.5 × 10^−3^ S/cm
poly(TT)1	2:1	3,300	4,000	1.21	56%	7.3 × 10^−6^ S/cm
poly(TT)2	4:1	3,700	4,500	1.22	88%	4.4 × 10^−5^ S/cm
poly(TT)3	8:1	3,500	4,200	1.24	90%	1.3 × 10^−4^ S/cm

**Figure 2:**
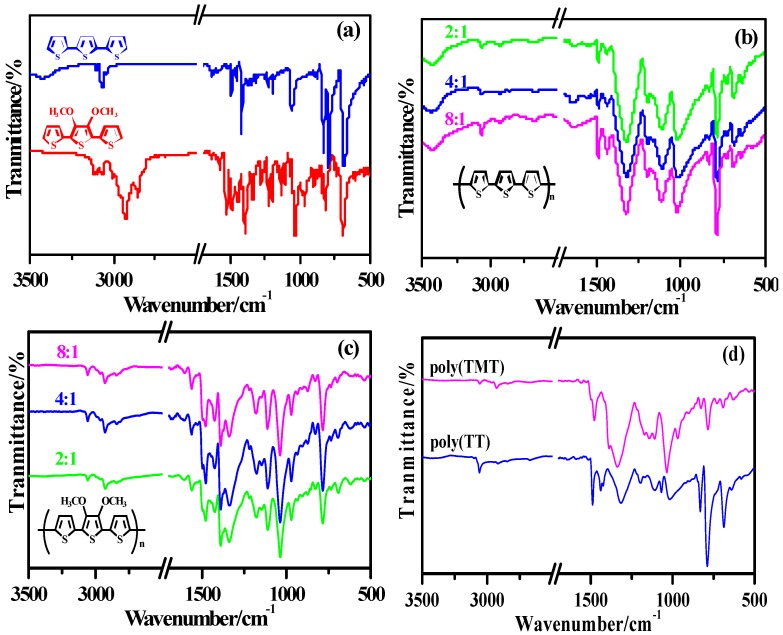
FT-IR spectra of monomers and polymers: (**a**) monomers (TMT and TT); (**b**) poly (TT)s; (**c**) poly (TMT)s; [FeCl_3_]/[monomer] ratio: 2:1; 4:1; 8:1; (**d**) undoped polymers ( [FeCl_3_]/[monomer] ratio: 8:1).

The comparison of the FT-IR spectra of monomers (TT and TMT), poly (TT)s, poly (TMT)s and undoped polymers ([FeCl_3_]/[monomer] ratio: 8:1) are presented in Figure 2. It is obvious from Figure 2b,c that poly (TMT)s present similar spectra to that of poly (TT)s, except for some bands associated with the introduction of dimethoxy substituent at ~2840–2927 cm^−1^, ~1389–1384 cm^−1^ and ~969–967 cm^−1^ [7,17]. From the spectra of poly (TMT)s and poly (TT)s, it can be observed that the intensive vibration bands appeared in TMT and TT at ~3100 cm^−1^ arising from α-H stretching of thiophene moiety disappear completely, whereas, the peaks at ~3060 cm^−1^assigned to the C***_β_***–H stretching mode are still remained in both poly(TT)s and poly(TMT)s. Furthermore, new strong intense bands at ~785 cm^−^^1^ due to the *β*-Hs of the thiophene rings appear in the polymer spectra [8,18,21], indicating that α,α'-bonding predominates in both poly(TT)s and poly(TMT)s. Furthermore, the intensity ratio of C–H***_β_*** to C–H***_α_*** deformation bands in poly(TMT) is almost the same as that of poly(TT), suggesting that the dimethoxy substituent has little influence on the α,α'-coupling. Comparing the spectra of poly- (TMT)s and poly(TET)s [12], it is found that *β*-defects in poly(TET)s are more than in poly(TMT)s. This can be related to the ethylene bridge, which can minimize steric distortion effects resulting in a high stereoregularity of TET, and consequently enhancing the formation of *β-*defects.

As can be seen from Figure 2d, the spectra of undoped polymers ([FeCl_3_]/[monomer] ratio: 8:1), such as: undoped poly(TT)3 and poly(TMT)3 are somewhat different from the respective as-prepared polymers. Further comparing the spectra of undoped and as-prepared polymers ([FeCl_3_]/[monomer] ratio: 8:1), one can see that the vibration bands appeared at ~1425 cm^−1^ and ~1390 cm^−1^ in as-prepared poly(TMT)3 almost disappeared in the case of undoped poly(TMT)3, while the intensity of vibration bands appeared at ~1320, 1120 and ~1027 cm^−1^ in as-prepared poly(TT)3 strongly decreased in the case of undoped poly(TT)3.All these observations suggests that the structure of as-prepared polymers ([FeCl_3_]/[monomer] ratio: 8:1) can be changed after dedoping, which is similar to the previous observation in case of dialkyl substituted poly(TT) [7].

### 2.2. UV-Vis Absorption Spectra

**Figure 3:**
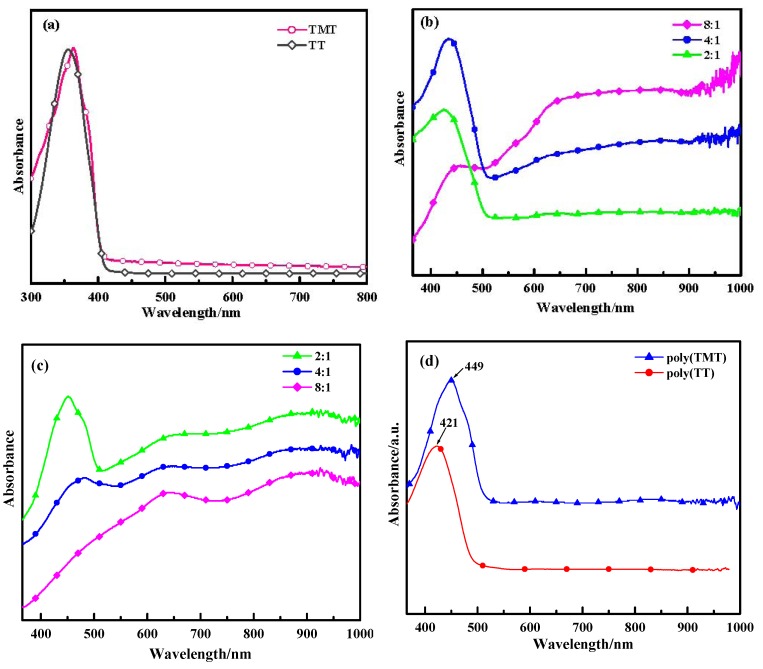
UV-vis of spectra of monomers and polymers: (**a**) monomers (TMT and TT); (**b**) poly(TT)s; (**c**) poly(TMT)s; [FeCl_3_]/[monomer] ratio: 2:1; 4:1; 8:1; (**d**) undoped polymers ([FeCl_3_]/[monomer] ratio: 8:1).

Figure 3 displays the optical absorption spectra of the monomers (TT and TMT), poly(TT)s, poly(TMT)s and undoped polymers ([FeCl_3_]/[monomer] ratio: 8:1) in acetonitrile. As shown in Figure 3a, TT and TMT show peaks at 355 nm and 364 nm, respectively. However, when comparing TT with TET (at 375 nm) [12], the bathochromic shift is obvious. This indicates that the effects of free dimethoxy units for enhancing the coplanirity of TT is smaller than those of ethylenedioxy units.

As depicted in Figure 3, poly(TMT)1 and poly(TMT)2 show three characteristic peaks at 451–483 nm, 650–662 nm and 910–918 nm, while poly(TMT)3 displays two characteristic peaks at 645 nm and 920 nm. In the case of poly(TT), poly(TT)s show two characteristic peaks at 426–457 nm and peaks at 640–660 nm with a free tail extending into the near infrared region. The peaks at 426–483 nm can be ascribed to the π→π* transitions of the polymer backbone, whereas the peaks at 640–662 nm and 910–920 nm can be attributed to polaron and/or bipolaron bands, which are characteristic of oxidized poly(TMT) and poly(TT) [23,24,25,26]. On comparison, increasing of the [FeCl_3_]/[TMT] ratio causes a decrease in the intensity of the π→π* absorption bands and a red-shift from 451 to 483 nm, accompanied with a progressively decreasing intensity of the peaks at 451–483 nm, especially π→π* absorption band disappears in the case of poly(TMT)3. In the case of poly(TT)s, the increasing [FeCl_3_]/[TT] ratio also causes a decrease in the intensity of the π→π* absorption bands and a red-shift from 426 to 457 nm, accompanied with the progressively increasing intensity of peaks at 640–660 nm with a free tail extending into the near infrared region. This implies that the oxidation degree of both of poly(TMT) and poly(TT) increases with the increasing [FeCl_3_]/[monomer] ratio.

Comparing the spectra of poly(TT)s and poly(TMT)s, a red shift for the peaks assigned to the π→π* transitions is observed in the spectra of poly(TMT)s, and the poly(TMT)s seem to be more oxidized than poly(TT) for the same [FeCl_3_]/[monomer] ratio, indicating enhanced stability in the oxidized state (doping state) of poly(TMT). This can be assigned to the strong electron-donating effect of the dimethoxy substituents, which will cause an increase in the conjugated length of the polymer chains [15,17].

In our previous report, we found that all the absorption spectra of poly(TET)s have maxima located at the same wavelength of 462 nm, ascribed to the π→π* transitions of the polymer backbone at different [FeCl_3_]/[TET] ratios [12], suggesting a red shift for the peak assigned to π→π* transitions compared with the reduced phase of poly(TMT). It is interesting that with its positive effect on the coplanirity of polymer backbone and similar electronic effects to dimethoxy groups, the ethylenedioxy group makes poly(TET)s more reduced than poly(TMT)s. The possible reason for this phenomenon may be related to the higher stability of the TET cation radical, which decreases the degree of polymerization. The lower degree of polymerization will cause a shorter polymer chain length in poly- (TET) than in poly(TMT) and poly(TT). Consequently, the lower degree of polymerization in poly(TET) hinders further doping, while the higher degree of polymerization in poly(TT)s and poly (TMT)s facilitates the degree of doping of the resulting polymer.

As a comparison, the spectra of undoped polymers ([FeCl_3_]/[monomer] ratio: 8:1), such as: undoped poly(TT)3 and poly(TMT)3 show only one characteristic peak at 421 and 449 nm, respectively, which are ascribed to the π→π* transitions of the polymer backbone, and the peaks attributed to polaron and/or bipolaron bands disappear after the dedoping. This phenomena is in good agreement with previous conclusions for methyl substituted poly(TT), in which the polaron and/or bipolaron bands disappeared after the electrochemically prepared methyl substituted poly(TT) was reduced at a potential of −0.4 V [26]. Furthermore, according to the earlier electrochemical and spectro-electrochemical studies on model oligothiophenes, it can be concluded that the spectra of poly(TT)s, and poly(TMT)s are characteristic for a sexithiophene dication [26].

### 2.3. XRD Patterns

**Figure 4:**
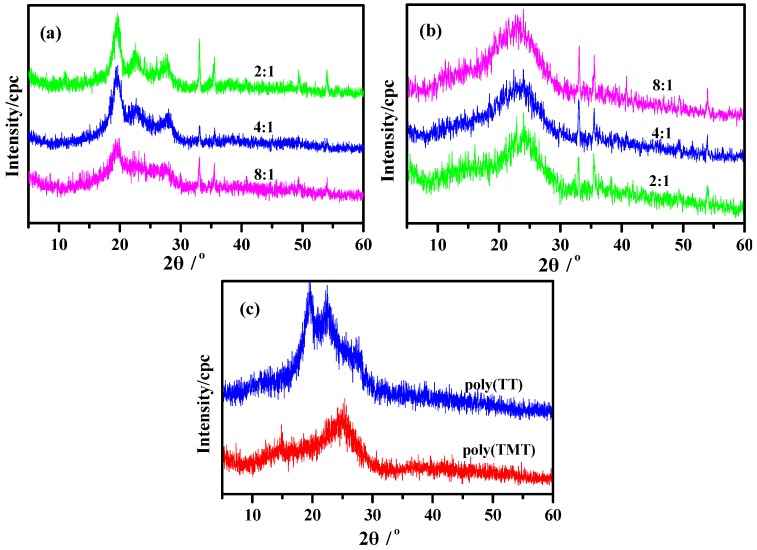
XRD of polymers: (**a**) poly(TT)s; (**b**) poly(TMT)s; [FeCl_3_]/[monomer] ratio: 2:1; 4:1; 8:1; (**c**) undoped polymers ([FeCl_3_]/[monomer] ratio: 8:1).

Figure 4 shows the powder XRD patterns of poly(TT)s, poly(TMT)s and undoped polymers ([FeCl_3_]/[monomer] ratio: 8:1). The powder XRD patterns of poly(TMT)s exhibit two broad diffraction peaks at approximately 2θ = 13°, 23°, respectively, associated to the intermolecular π→π* stacking or assigned to the (200) and (020) reflection [15,27,28]. As shown in Figure 4a, poly(TT)s present a highly crystalline structure which is similar to the poly(***α***-quaterthiophene) and chemically coupled poly(thiophene) [27]. All poly(TT)s display three main diffraction peaks at 2θ = ~19°, ~23°, ~27°, respectively, which are the 110, 200, and 210 reflections of the crystalline fraction of sample according to previous research [27,28]. It is clear from Figure 4, that aside from the main diffraction peaks, the sharp diffraction peaks at 2θ = 33°, 35°, 49°, 54° with low intensity which are present in all poly- (TT)s and poly(TMT)s correspond to the FeC1_4_^−^ doping agent, which generally accompanies the as-made polythiophene [12].

Figure 4 also implies that the poly(TMT)s display more amorphous than poly(TT)s, and there are no big differences in XRD patterns while varying the [FeCl_3_]/[TMT] ratio (Figure 4b). This can be associated with the introduction of dimethoxy substituents in the terthienyl rings, which may decrease the crystallinity with its separation effect on the polymer main chains. This phenomenan was also observed while comparing the XRD results of highly crystalline polythiophene [28] with poly(EDOT) [15]. It is clear from the comparison of XRD patterns of poly(TMT)s and poly(TET)s [12], that the diffraction peaks associated to the intermolecular π→π* stacking are shifted to a lower angle and are more amorphous, which may result from the increased interchain spacing introduced by the free dimethoxy substituents.

In comparison to the as-prepared polymers ([FeCl_3_]/[monomer] ratio: 8:1), in the powder XRD patterns of undoped poly(TT)3 and poly(TMT)3, the sharp diffraction peaks at 2θ = 33°, 35°, 49°, 54° with low intensity presented in all poly(TT)s and poly(TMT)s corresponding to the FeC1_4_^−^ doping agent almost disappeared in the case of undoped poly(TT)3 and poly(TMT)3. Furthermore, the crystallinities of the undoped poly(TT)3 and poly(TMT)3 are lower than those of the respective as-prepared polymers, which indicates that the dedoping process can make the polymers more amorphous than as-prepared polymer.

### 2.4. Thermal Stability

**Figure 5:**
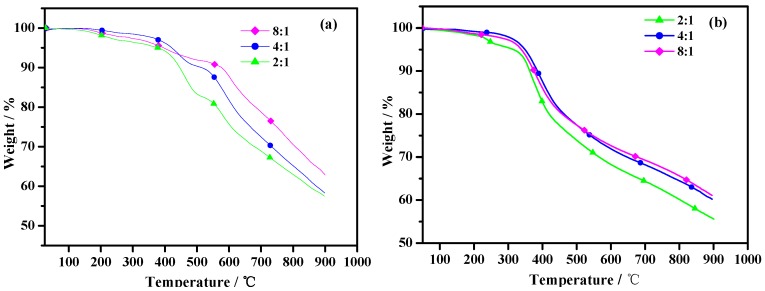
TGA of polymers: (**a**) poly(TT)s; (**b**) poly(TMT)s; [FeCl_3_]/[monomer] ratio: 2:1; 4:1; 8:1.

**Figure 6:**
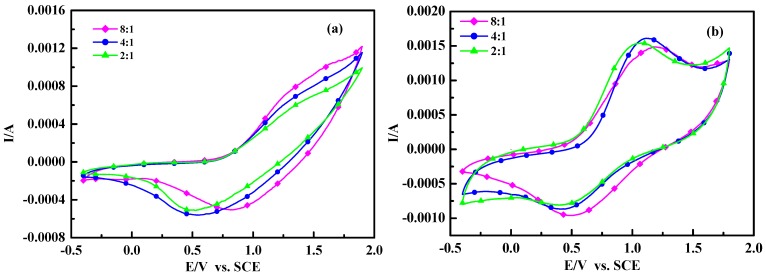
Cyclic voltammograms of polymers: (**a**) poly(TT)s; (**b**) poly(TMT)s; [FeCl_3_]/[monomer] ratio: 2:1; 4:1; 8:1.

Figure 5 represents the weight (%) loss versus temperature data for poly(TT)s and poly(TMT)s. As shown in Figure 6, poly(TT)s and poly(TMT)s display three major weight loss steps. The initial weight loss of less than 5% is due to the volatilization of solvents, and/or absorbed moisture, and oligomers of the monomer. Comparing the TGA curves of poly(TMT)s and poly(TT)s, it can be observed that the initial degradation temperature (384–421 °C) of poly(TT)s is higher than that of poly(TMT)s (315–347 °C), indicating that the poly(TT) exhibits higher thermal stability than poly(TMT). The improved stability of poly(TT) as compared to poly(TMT), can be attributed to its highly symmetric structure and the absence of any alkyl or alkoxy substituents in the terthienyl chain, which is generally considered as a main factor decreasing the thermal stability of the polythiophene [21]. Furthermore, poly(TMT)s exhibit lower thermal stability than poly(TET) [12]; the reason is possibly the lower crystallinity of poly(TMT)s, and consequently this decreases the thermal stability of the polymer.

As shown in Figure 5, the initial weight loss temperatures for poly(TMT)s are found to increase in the order of poly(TMT)1 < poly(TMT)3 < poly(TMT)2, and the same order is also observed in the case of poly(TT)s. By comparison, in the case of poly(TT)s and poly(TMT)s, the polymers prepared using the highest [FeCl_3_]/[monomer] ratio give the highest residue at 900 °C, which can result from a higher conjugated length of polymers than that of others. However, in the case of poly(TET) [12], there is only slight difference in TGA curves, suggesting that [FeCl_3_]/[TET] ratio has little effect on thermal stability of poly(TET). This is in agreement with the results from UV-spectra of poly(TET)s, indicating the same structural properties in poly(TET)s resulted from the low molecular weight and doping degree, as well as the slight differences in the molecular weight in poly(TET)s. 

### 2.5. Electrochemical Properties and Conductivity

Figure 6 shows the cyclic voltammograms (CVs) of poly(TT)s and poly(TMT)s films on Pt in 0.1 mol/L Et_4_NBF_4_acetonitrile solution at a scan rate of 50 mV/s. As seen in Figure 6, the anodic peaks (~1.5–1.6 V) of poly (TT)s are not clearly defined, while well defined cathodic peaks are located at 0.5–0.8 V. The CVs of poly(TT)s are very similar to those of electrochemically synthesized poly(TT) [20,25,26].

In the case of poly(TMT)s, well defined anodic and cathodic peak potentials are observed at 1.1–1.21 V and 0.4–0.5 V, respectively. It is obvious from the comparison of CVs of poly(TT)s and poly(TMT)s, that the electrochemical windows of poly(TMT)s are larger than those of poly(TT)s, suggesting higher coulombic capacity, and the electrochemical oxidation of poly(TMT)s started at 0.55–0.63 V, which is lower than that of poly (TT)s (0.72–0.79 V). However, the electrochemical oxidation of poly(TMT)s started at 0.55–0.63 V, which is lower than that of poly(TET)s (0.21–0.44 V) [12].

All these results show that the poly(TMT)s are electrochemically more active than poly(TT)s, but less active than poly(TET)s. This was further proven by conductivity measurements. The conductivity measurements reveal that the as-made poly(TMT)s and poly(TET)s [12] have three orders of magnitude lower conductivity at room temperature, while poly(TT)s shows lower conductivity than that of poly(TMT)s (Table 1). The differences in conductivities of poly(TMT)s and poly(TET)s can be understood by the more compact and rigid structure in polymer chains of poly(TET)s than in poly(TMT). Although the free dimethoxy substituents can facilitate the doping process and result in less β-defects, the steric distortion effects of the dimethoxy substituents decreases the co-planarity of the polymer chains, and consequently make the poly(TMT) more amorphous with decreased conductivity and electrochemical activity than poly(TET). As shown in Table 1, the conductivities of poly(TT)s and poly(TMT)s increase with the increasing [FeCl_3_]/[monomer] ratio. This can be explained by the UV-spectra results, which indicate that the oxidation degree of poly(TT) and poly(TMT) increase steadily with the increasing of [FeCl_3_]/[monomer] ratio, consequently the higher oxidation degree will produce higher conductivity in the polymers. As a comparison, Zak *et al.* have reported the electrochemistry of the oligothiophenes [sexi (3-octylthiophene) oligomer] in solid state, and the solid film of sexi(3-octyl thiophene) on electrode undergoes multiple redox processes [29]. Jadamiec *et al.* have reported that a well defined peak at 0.49 V was observed for oligo-3'-methyl-2,2':5',2"-terthiophene during the electrochemical polymerization of 3'-methyl-2,2':5',2"-terthiophene [26]. However, in this case, the cyclic voltammograms (CVs) of poly(TMT) or poly(TT) do not show well defined sharp peaks for oligoterthiophene. This phenomena may be the result of the special components of these polymers. As concluded from the ^1^H-MNR and UV-vis absorption spectra results of, poly(TMT) or poly(TT) are mostly dimers of TMT or TT with a small amount of higher molecular weight components (trimer or longer oligomers). Generallycharge transfer is the main factor during the redox process for the appearance of defined sharp peaks in CVs. In this case, the charge transfer during the redox process could be restricted by the lower conductivity of higher molecular weight components in these polymers. Consequently, the redox process of oligo-terthiophene are difficult.

## 3. Experimental

### 3.1. General

Fourier transform infrared (FT-IR) spectra were recorded on a Bruker Equinox-55 FT-IR spectrometer using KBr pellets. The ^1^H-NMR and ^13^C-NMR spectra (400 MHz) were obtained using a Mercury-VX300 spectrometer. UV-vis spectra were obtained using a Hitachi-U3010 spectrophotometer. Thermal analysis was performed with an Netzsch STM90 thermal analysis instrument; the samples were heated from 25 °C to 900 °C at a rate of 10 °C min^−1^ under a nitrogen atmosphere, at a flow rate of 100 mL min^−1^. X-ray diffraction (XRD) patterns were recorded with an D/Max 2400 X-ray diffractometer using Cu Ka radiation, 40 kV, 40 mA. GPC (gel permeation chromatography) measurements were performed on a Waters GPCV-2000 apparatus equipped with a RI detector using DMF as the solvent at a rate of 1.0 mL/min, twenty microliters of 1.0% (w/v) sample solutions were injected for each analysis, calibration was accomplished with polystyrene standards (Polysciences, USA). Reagent grade terthiophene, 3,4-dimethoxythiophene, 2-(tributylstannyl)-thiophene, *N*-bromo-succinimide (NBS) and anhydrous iron (III) chloride were obtained from Aldrich. 2,5-Dibromo-3,4-dimethoxythiophene and Pd(PPh_3_)_4_ were synthesized according to the literature [13,14]. Other chemicals used were of analytical reagent grade, and used as received without further treatment. 

### 3.2. Synthesis of TMT

**Scheme 1:**
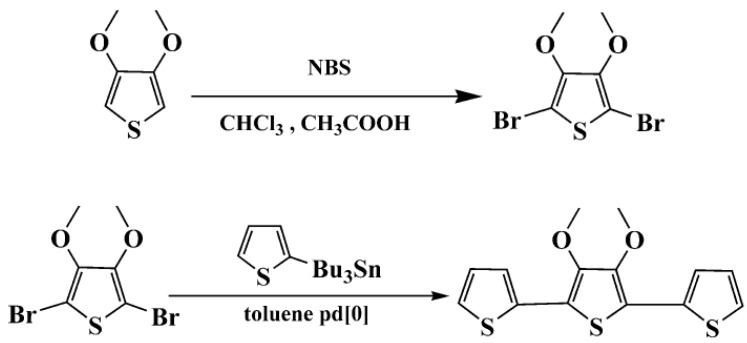
Synthesis of 3',4'-dimethoxy-2,2':5', 2"-terthiophene (TMT).

3',4'-Dimethoxy-2,2':5',2"-terthiophene (TMT) was synthesized according to the procedure given in [12], as shown in Scheme 1. ^1^H-NMR (DMSO-d_6_, ppm): δ: 7.58 (dd, 2H, *J* = 5.1 Hz, 1.1 Hz), 7.30 (dd, 2H, *J* = 3.6, 1.1Hz), 7.11 (dd, 2H, *J* = 5.1 Hz, 3.6 Hz), 3.90 (s, 4H). ^13^C-NMR (DMSO-d_6_, ppm): 121.97, 128.68, 132.32, 132.59, 138.25, 150.71. ^1^H-NMR and ^13^C-NMR spectra are shown in Figure 1.

### 3.3. Solid-State Polymerization of TMT and TT

A typical solid-state polymerization procedure was as follows [12]: 3',4'-Dimethoxy-2,2':5',2"-terthiophene (TMT, 0.308 g, 0.001 mol) and anhydrous iron (III) chloride (FeCl_3_, 0.324 g, 0.002 mol) were put in a mortar at room temperature, are ground to mix them thoroughly. After grinding the reactant about 30 min, the mixture became a pale green solid, then by further grinding for 2.5 h, the color of the mixture changed to black. The resulting polymer was removed from the solid mixture by repeatedly washing with ether, ethanol and distilled water until the filtrate was colorless, and then the powder was dried under vacuum at 50 °C for 48 h. The obtained polymer was designated as poly(TMT)1. In similar manner, the molar ratio of oxidant to monomer (represented by [FeCl_3_]/[TMT]) was adjusted at 4:1 and 8:1, respectively, the resulting polymers were noted as poly (TMT)2 and poly (TMT)3.

The poly (TT)s (Poly (TT)1, poly (TT)2, poly (TT)3) were synthesized in similar manner by adjusting [FeCl_3_]/[TT]) ratio at 2:1, 4:1 and 8:1, respectively. The poly(TMT)3 and poly(TT)3 were treated with reducing agent (hydrazine monohydrate, N_2_H_4_ × H_2_O) for 24 h at room temperature under nitrogen to give undoped polymers.

### 3.4. Electrochemical Tests

The cyclic voltammetry (CV) was performed in a conventional three-electrode cell using CHI660A electrochemical workstation, the working electrode was a polymer film electrode prepared by casting a DMF solution of polymer on a platinium electrode. The reference electrode was SCE, and the counter electrode was a 1 cm^2^ area Pt flag, and the supporting electrolyte was 0.1 mol/L tetraethylammonium tetrafluoroborate (Et_4_NBF_4_)/acetonitrile solution. The conductivity of polymers was measured by using the standard four-probe technique.

## 4. Conclusions

In this work, a new poly(terthiophene) derivative: poly (3',4'-dimethoxy-2,2':5',2"-terthiophene) was synthesized by a solid-state oxidative polymerization method at various ratios of FeCl_3_ to TMT. The experimental results were compared with those of polyterthiophene [poly(TT)] and poly(3',4'-ethylenedioxy-2,2':5',2"-terthiophene) [poly(TET)] prepared under the same solid-state oxidative polymerization conditions. The comparison results suggested that the dimethoxy substituents were a main factor for the structural and physicochemical differences between the poly(TMT), poly(TT) and poly(TET). Although the free dimethoxy substituents can facilitate the doping process and produce less β-defects, the steric distortion effects of dimethoxy substituents led the poly(TMT) to become more amorphous and less thermally stable with decreased conductivity and electrochemical activity than poly(TET). Furthermore, the steric distortion effects of dimethoxy substituents led the poly(TMT) to have a similar change tendency as poly(TT), in which the oxidation degree, electrochemical activity and conductivity increased steadily with increasing [FeCl_3_]/[monomer] ratio. In addition, the poly(TMT) or poly(TT) are mostly a dimer of TMT or TT with a small amount of higher molecular weight components (trimer or longer oligomers). Well defined sharp peaks for oligoterthiophene was not observed due to the restriction of charge transfer during the redox process by the lower conductivity of higher molecular weight components in these polymers.
